# Virus-Like Particle Based Vaccines Elicit Neutralizing Antibodies against the HIV-1 Fusion Peptide

**DOI:** 10.3390/vaccines8040765

**Published:** 2020-12-15

**Authors:** Alemu Tekewe Mogus, Lihong Liu, Manxue Jia, Diane T. Ajayi, Kai Xu, Rui Kong, Jing Huang, Jian Yu, Peter D. Kwong, John R. Mascola, David D. Ho, Moriya Tsuji, Bryce Chackerian

**Affiliations:** 1 Department of Molecular Genetics and Microbiology, University of New Mexico, Albuquerque, NM 87131, USA; amogus@salud.unm.edu (A.T.M.); DtAdebanjo@salud.unm.edu (D.T.A.); 2 Aaron Diamond AIDS Research Center, New York, NY 10016, USA; ll3411@cumc.columbia.edu (L.L.); manxue.jia@adlainortye.com (M.J.); jh4236@cumc.columbia.edu (J.H.); jy3068@cumc.columbia.edu (J.Y.); dh2994@cumc.columbia.edu (D.D.H.); 3 Department of Medicine, Columbia University Irving Medical Center, New York, NY 10032, USA; 4 Vaccine Research Center, NIAID, NIH, 40 Convent Drive, Bethesda, MD 20892, USA; kai.xu@nih.gov (K.X.); rui.kong@nih.gov (R.K.); pdkwong@nih.gov (P.D.K.); jmascola@nih.gov (J.R.M.)

**Keywords:** broadly neutralizing antibody, HIV-1 fusion peptide, HIV-1 DS-SOSIP trimer, prime-boost immunizations, vaccine, virus-like particles

## Abstract

Broadly neutralizing antibodies (bnAbs) isolated from HIV-infected individuals delineate vulnerable sites on the HIV envelope glycoprotein that are potential vaccine targets. A linear epitope within the N-terminal region of the HIV-1 fusion peptide (FP8) is the primary target of VRC34.01, a bnAb that neutralizes ~50% of primary HIV isolates. FP8 has attracted attention as a potential HIV vaccine target because it is a simple linear epitope. Here, platform technologies based on RNA bacteriophage virus-like particles (VLPs) were used to develop multivalent vaccines targeting the FP8 epitope. Both recombinant MS2 VLPs displaying the FP8 peptide and Qβ VLPs displaying chemically conjugated FP8 peptide induced high titers of FP8-specific antibodies in mice. Moreover, a heterologous prime-boost-boost regimen employing the two FP8-VLP vaccines and native envelope trimer was the most effective approach for eliciting HIV-1 neutralizing antibodies. Given the potent immunogenicity of VLP-based vaccines, this vaccination strategy—inspired by bnAb-guided epitope mapping, VLP bioengineering, and prime-boost immunization approaches—may be a useful strategy for eliciting bnAb responses against HIV.

## 1. Introduction

Human immunodeficiency virus type 1 (HIV-1) continues to impose a significant burden of disease worldwide. Despite some progress in animal models [1,2,3], candidate vaccines evaluated to date have not yet been successful in inducing immunity in humans that protects from HIV infection. There is widespread agreement that the development of a successful HIV-1 vaccine will be dependent on the ability to induce potent protective antibodies capable of neutralizing diverse HIV-1 isolates. These antibodies, termed broadly neutralizing antibodies (bnAbs), are found in nearly 50% of HIV-infected individuals, but only after multiple rounds of immune selection and viral escape [4,5]. BnAbs commonly target a few sites of vulnerability on the HIV envelope glycoprotein (or Env trimer): the CD4 binding site [5,6], the trimer V1V2 apex [7,8], the variable glycan-V3 loop [9,10], the gp120-gp41 interface, the fusion peptide, and the membrane-proximal external region [11,12]. The isolation of bnAbs from HIV-infected patients and identification of their target epitopes on the conserved regions of HIV-1 Env trimer have provided possible pathways for vaccine design. However, many bnAbs recognize complex conformational epitopes or have undergone extensive affinity maturation from their germline state. These factors have complicated schemes for devising effective vaccine regimens for eliciting bnAb responses [13].

The HIV-1 fusion peptide is a critical component of the viral entry machinery that is composed of 15-20 hydrophobic residues at the N-terminus of the gp41 subunit of HIV-1 Env [12]. A simple linear epitope consisting of 8 amino acids at the N-terminus of the gp41 fusion peptide (called FP8) has been shown to be a primary target of a bnAb, VRC34.01, derived from an HIV-patient [12,14]. Recently, Xu et al. designed an FP8-based vaccine in which the peptide is conjugated to keyhole limpet hemocyanin (KLH) through maleimide linkage chemistry [3]. This FP8-KLH vaccine elicited fusion peptide-directed antibodies in mice, guinea pigs, and rhesus macaques that were capable of neutralizing diverse strains of HIV-1 [3]. Intriguingly, this FP8-KLH vaccine, in combination with multiple Env trimer boosts, elicited FP8-directed antibodies in rhesus macaques that neutralized 59% of 208 diverse viral strains [15].

Antibodies targeting FP8 that have been elicited by infection or by vaccination exhibit broad neutralization [3,12,15]; however, neutralization activity against specific HIV-1 isolates is dependent on the structural diversity of the fusion peptide (FP), sensitivity to mutations at specific antibody interaction sites of the Env trimer outside of the FP and the natural diversity in FP8 sequences [14,16]. FP, in the context of the native HIV Env trimer, adopts multiple conformations and orientations, which can both facilitate and complicate recognition of the FP8 epitope by bnAbs [12,16,17]. X-ray crystallography and cryo-electron microscopy of the FP8-targeting antibodies in complex with the FP and Env trimer revealed diverse modes of antibody recognition of FP8 on the native HIV Env glycoprotein [12,16]. Known FP8-targeting bnAbs approach the HIV Env trimer from different angles and recognize this epitope by penetrating through a highly conserved glycosylation site on gp120 (N88) [16]. Mutations within this region of gp120 potentially affect the neutralization breadth of FP8-specific bnAbs [14,16]. In addition, FP8 is not completely conserved in sequence across different subtypes of the HIV-1 Env [12,14]. Functional mapping of infection- and vaccine-elicited antibodies against the FP has identified escape mutants and common variant sequences within the FP8 epitope [14]. While functional mapping likely helps to re-center the protective domain on newly emerging HIV strains for subsequent rounds of immunogen design, the inherent structural diversity of FP may enable the design of conformationally diverse FP8 immunogens using multiple carrier protein or virus-like particle platforms, capable of eliciting antibodies to recognize and neutralize FP in the context of native Env trimer.

Virus-like particles (VLPs) are a safe and highly immunogenic class of vaccines that have been utilized in many pre-clinical, clinical, and post-clinical studies, with approved human vaccines available against hepatitis B virus [18], human papillomavirus [19], and hepatitis E virus [20]. There has been increasing interest in bioengineering VLPs to serve as highly immunogenic platforms for surface display of foreign epitopes or antigens in a multivalent architecture [21,22,23,24]. Bacteriophage VLPs are a particularly flexible and modular platform for vaccine development allowing the display of heterologous antigens on the surface of VLPs in a highly dense, repetitive array by several different techniques [22,25]. For example, MS2 and PP7 bacteriophage coat protein single-chain dimers have been engineered for genetic insertion of heterologous peptides, and to produce in vivo assembled VLPs displaying heterologous peptides using bacterial cell factories [26,27]. These recombinant VLPs are highly immunogenic and confer high immunogenicity to heterologous peptides displayed on their surfaces [22,28]. In addition, bacteriophage VLPs can be chemically modified to display target antigens. For example, some bacteriophage VLPs, including Qβ, contain a high density of surface-exposed lysines which can be targeted for modification using various chemical techniques, enabling multivalent display of diverse target antigens [29]. Qβ VLPs are produced by recombinant expression of the Qβ coat protein in bacteria and by subsequent in vivo self-assembly of 180 monomers into VLPs. Target peptides, which have been synthesized to contain a free terminal cysteine, can then be conjugated to the VLP using an amine- and sulfhydryl-reactive bifunctional cross-linker. Qβ VLP based vaccines targeting antigens from both pathogens and self-antigens have been constructed and assessed in numerous preclinical studies [27,30,31], as well as human clinical trials [32,33,34].

In this study, we describe using both chemical conjugation and genetic insertion to construct microbially synthesized RNA bacteriophage VLPs displaying the HIV-1 FP8 epitope. Both approaches enable multivalent display of the FP8 epitope on the surface of *Escherichia coli* produced bacteriophage VLPs. Both conjugated Qβ-FP8 and recombinant MS2-FP8 VLPs were recovered with high purity, and the recombinant VLPs maintained their in vivo assembly capacity. The FP8-VLPs were tested in different prime-boost regimens to elicit FP8-specific HIV-1 neutralizing antibody in mice. In particular, IgG isolated from mice immunized with an MS2-FP8 VLP prime, and boosts with Qβ-FP8 VLPs and native trimeric Env (BG505 DS-SOSIP), had the strongest neutralizing activity against prototype Clade A and B HIV-1 virus isolates. These studies suggest that VLP-based vaccines could be a useful component of a heterologous FP8 targeted vaccine strategy for eliciting bnAbs against HIV-1.

## 2. Materials and Methods

### 2.1. Construction of FP8-Displaying Recombinant VLPs

Plasmid pDSP62, which encodes the single-chain dimer version of the MS2 bacteriophage coat protein, was generated previously [35]. The gene fragment encoding FP8 with as well as sequence encoding a flanking Ser-Thr-Gly-Val-Gly-Ser (STGVGS) peptide linker sequence was cloned at the 5′ end of the single-chain dimer sequence by PCR. Briefly, a forward PCR primer (5’ GCGCCATGGCAGCGGTTGGCATTGGAGCAGTTTTCTCAACCGGAGTTGGAAGCGCAAGCAATTTCACGCAATTTG 3’) was designed to contain nucleotide sequence encoding a *NcoI* restriction site, a start codon and an alanine amino acid, the FP8 sequence, a linker sequence and a sequence that is complementary to the N-terminus of the MS2 single-chain dimer coat protein. The reverse primer E3.2 (5’ CGGGCTTTGTTAGCAGCCG G 3’) that anneals downstream of a unique *BamHI* site in the pDSP62 plasmid, was described previously [27]. The primers were used to amplify a gene fragment using plasmid pDSP62 as a PCR template DNA. Amplified MS2-FP8 PCR fragment was digested with *NcoI* and *BamHI* restriction enzymes and cloned into pDSP62 plasmid using these restriction sites. The cloned construct was sequenced to confirm the presence of the FP8 peptide insert and designated as pDSP62-FP8.

### 2.2. Production and Purification of FP8-Displaying Recombinant VLPs

pDSP62-FP8 plasmids were transformed into C41 *E. coli* cells by electroporation. Transformed C41 cells were grown at 37 °C using Luria Bertani broth containing 60 µg/mL kanamycin until the cells reached an OD600 of 0.6. MS2-FP8 protein expression was induced using 0.4 mM isopropyl-β-D-1-thiogalactopyranoside and grown at 37 °C overnight. Cell pellets were collected and re-suspended using a lysis buffer [50 mM Tris-HCL, 100 mM NaCl, 10 mM ethylenediaminetetraacetic acid, pH 8.5]. Cells were lysed by sonication and cell lysates were clarified by centrifugation (15,000× *g*, 20 min, 4 °C). Soluble MS2-FP8 VLPs were purified by selective-salting out precipitation using 70% saturated (NH_4_)_2_SO_4_, followed by an additional polishing size exclusion chromatography (SEC) step using a Sepharose CL-4B column. The column was pre-equilibrated with a purification buffer (40 mM Tris-HCl, 400 mM NaCl, 8.2 mM MgSO_4_, pH 7.4). MS2-FP8 VLPs were concentrated from SEC purified fractions by ultra-centrifugal filtration using Amicon^®^ Ultra-15.0mL 100 K membrane (Merck Millipore Ltd., Tullagreen, Carrigtwohill, Co. Cork, Ireland; 3000g, 10 min at 22 °C).

### 2.3. Conjugation of FP8 to Qβ Bacteriophage VLPs

Qβ-VLPs were produced in *E. coli* using methods as previously described to produce MS2 bacteriophage VLPs [27]. The FP8 peptide (AVGIGAVF) was synthesized (GenScript, Piscataway, NJ, USA) and modified to include a C-terminal cysteine residue preceded by a 3-glycine-spacer sequence (-GGGC). FP8-GGGC peptides were conjugated to Qβ-VLPs using the bifunctional cross-linker succinimidyl 6-β-maleimidopropionamido hexanoate (SMPH) (Thermo Fisher Scientific, Waltham, MA, USA) as described previously for the conjugation of amyloid-beta (Aβ) peptides [36].

### 2.4. Characterization of FP8-Displaying VLPs

Conjugated (Qβ-FP8) and recombinant (MS2-FP8) VLPs were run on a 10% sodium dodecyl sulfate polyacrylamide gel electrophoresis (SDS-PAGE) gel stained with Coomassie blue to check the efficiency of conjugation and the purity of the VLPs. All VLP concentrations were estimated from SDS-PAGE gel protein bands corresponding to Qβ, Qβ-FP8, MS2, and MS2-FP8 coat proteins using known amounts of hen egg lysozyme as the standard protein. Visualization of the VLPs with transmission electron microscope (TEM) was performed as previously reported [37]. Briefly, VLPs were adsorbed on carbon-coated glow-discharged copper grids for 2 minutes, and were negatively stained with 2% uranyl acetate for 2 minutes. VLPs were visualized with a TEM at a magnification of 200,000X. The display of FP8 peptides on the surface of Qβ and MS2 VLPs was confirmed by enzyme-linked immunosorbent assay (ELISA) as follows: the 96-well Immulon^®^ 2 plate (Thermo Fischer Scientific) was coated with 500 ng of Qβ-FP8 VLPs, MS2-FP8 VLPs, and negative controls (unmodified Qβ and MS2 VLPs) in duplicate diluted in 50 µL of phosphate-buffered saline (PBS) in each well. The plate was incubated at 4 °C for overnight and blocked for 1 h at room temperature with blocking buffer (0.5% dry milk in PBS). Wells were washed two times with PBS and incubated for 2 h at room temperature with dilutions of VRC34.01 in blocking buffer. VRC34.01 was expressed in 293 F cells by transient transfection using plasmids coding for the heavy and light chains of the antibody, as previously described [12]. The wells were washed five times with PBS and probed with horseradish peroxidase (HRP)-conjugated secondary antibody (goat anti-human IgG (Jackson ImmunoResearch, West Grove, PA, USA; 1:5000)) for 1 h. The reaction was developed using TMB (Thermo Fischer Scientific) and stopped using 1% HCl. Reactivity of VRC34.01 for the target FP8 peptides was determined by measuring optical density at 450 nm (OD_450_).

### 2.5. Mice Immunizations

All animal studies were performed in accordance with guidelines of the University of New Mexico Animal Care and Use Committee (Protocol #: 19-200870-HSC). Seven groups of five female BALB/c mice, aged 6–8 weeks, were obtained from Jackson Laboratory (Bar Harbor, ME, USA). All groups of mice were vaccinated three times (with prime, first and second booster doses) intramuscularly at three-week intervals (at weeks 0, 3, and 6). VLPs were immunized at a dose of 5 µg without exogenous adjuvant. At the second boost, some groups were immunized with 25 µg of BG505 DS-SOSIP.664 and formulated with Alhydrogel^®^ (InvivoGen, San Diego, CA, USA) at a 1:1 (volume:volume) ratio. BG505 DS-SOSIP.664 was produced from a CHO-DG44 stable cell line, purified using non-affinity chromatography and was antigenically similar to trimers described previously [38]. The injection volume was 50 µL for all formulations. Blood was collected two weeks following the first and second boosts (at weeks 5 and 8); serum was isolated and stored at −20 °C.

### 2.6. Characterization of Antibody Responses

FP8- and BG505 DS-SOSIP-specific mice serum IgG titers were determined by end-point dilution ELISA. For FP8 peptide ELISAs, Immulon^®^ 2 plates (Thermo Fisher Scientific) were incubated with 500 ng streptavidin (Invitrogen) in PBS, pH 7.4, for 2 h at 37 °C. Following washing, SMPH was added to wells at 1.0 µg/well and incubated for 1 h at room temperature. The FP8 peptide was added to the wells at 1.0 µg/well and incubated overnight at 4 °C. BG505 DS-SOSIP ELISAs were performed based on a previously reported lectin captured trimer method, with slight modifications [39]. The plates were coated with 50 µL/well of 2.0 µg/mL of Lectin (*Galanthus nivalis)* (Sigma Aldrich, St. Louis, MO, USA) in PBS overnight at 4 °C. After blocking, 50 µL/well of 2.0 µg/mL BG505 DS-SOSIP in blocking buffer was added and incubated for 2 h at room temperature. For all ELISAs, plates were blocked with 0.5% milk in PBS (150 µL/well) for 1 h at room temperature, and four-fold dilutions of mice sera (starting at 1:40) were added to each well and incubated for 2 h. The wells were probed with horseradish peroxidase (HRP)-conjugated secondary antibody [goat anti-mouse-IgG (Jackson Immuno-Research; 1:5000)] for 1 h. The reaction was developed using TMB (Thermo Fischer Scientific) and stopped using 1% HCl. Reactivity of sera for the target antigen was determined by measuring OD_450_. Wells with twice the OD_450_ value of background were considered positive; the highest dilution with a positive value was considered as the end-point dilution titer.

### 2.7. Immunoglobulin Purification

Dynabeads^TM^ Protein G (Invitrogen, Carlsbad, CA, USA) were used to purify IgG from pooled mice sera from selected immunized groups (Groups II, III, and VII). First, the Dynabeads^TM^ Protein G were washed with PBS by placing the tube in a DynaMag™ magnet (Thermo Fischer Scientific). The mice sera were incubated with the Dynabeads^TM^ Protein G in the tubes for 40 minutes at room temperature. The Dynabeads were washed with PBS by placing the tubes in the DynaMag™ magnet to remove unbound materials. IgG was eluted using 0.2 M glycine/HCl buffer (pH 2.7) and then brought to neutral pH using 1 M Tris buffer (pH 9). The concentration of purified mouse IgG was measured by reading absorbance at 280 nm.

### 2.8. HIV-1 Neutralization Assay

The neutralization activity of pooled sera or purified IgG from BALB/c mice immunized with various prime-boost-boost compositions was determined by using the TZM-bl assay. Sera were assessed in duplicate at eight 4-fold dilutions, starting at a 1:20 dilution. Sera were incubated with HIV-1 Q23.17 (Clade A) virus prior to adding to TZM-bl cells and incubated for 3 days. After removing supernatant, the cells were lysed, and β-Gal was added to detect the β-galactosidase activity, and a 50% inhibitory concentration (IC_50_) were determined. The IC_50_ were expressed as the reciprocal of the serum dilutions required to inhibit infection by 50%. Serum IgGs were purified (Section 2.7 above) from the immune sera (Group II and Group III mice) capable of inhibiting infection by 50%, as well as from control (Group VII) mice. Purified serum IgGs were tested against Q23.17 (Clade A), BG505 (Clade A) and BaL.01 (Clade B) HIV-1 virus isolates in triplicate using seven 4-fold dilutions, starting at 50 μg/mL. Data are reported as percent (%) neutralization inhibition. Purified IgG from Group VII mice and VRC34.01 bnAb were used as negative and positive controls, respectively.

### 2.9. Statistical Analysis

Statistical analysis was carried out using GraphPad Prism Version 6.00 (GraphPad Software Inc., San Diego, CA, USA). Comparison between two groups was performed with paired t test or unpaired two-tailed t test. Groups were considered significantly different (*) at *p* < 0.05.

## 3. Results and Discussion

### 3.1. Engineering and Characterization of FP8-Displaying VLPs

**Figure 1:**
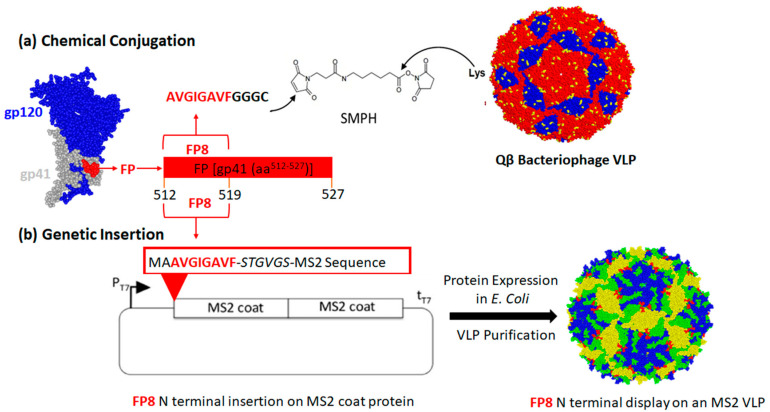
Strategy for displaying the HIV-1 fusion peptide epitope (FP8) on bacteriophage (virus-like particles, VLPs) (**a**) by chemical conjugation or (**b**) by constructing recombinant VLPs. In (a), the FP8 peptide was synthesized to contain a linker sequence with a terminal cysteine residue (-GGGC). The peptide was then conjugated to the surface-exposed lysine (Lys, in yellow) residues on microbially synthesized and in vivo assembled Qß bacteriophage VLPs (dimers formed from A- and B-chains in red, and C-chain homodimers in blue) using a bifunctional crosslinker (SMPH). In (b), DNA sequence encoding the FP8 peptide was inserted into the N-terminus of MS2 bacteriophage coat protein single-chain dimer sequence on an expression vector. Recombinant MS2 VLPs displaying FP8 peptides were expressed from a plasmid in *E. coli*. The green, blue, and yellow colors on MS2 VLP structure in the figure represent the residues derived from A-, B- and C-chains, respectively. The red color indicates the location of the N-terminal insertion site.

Modification of the exterior facets of bacteriophage VLPs by genetic insertion or chemical conjugation techniques has enabled multivalent display of diverse heterologous epitopes on the surface of VLPs [21]. We previously showed that an engineered version of the MS2 bacteriophage coat protein, called the single-chain dimer, can be used to display target peptides on the surface of VLPs, either at a constrained loop (the AB-loop) [26] or at the N-terminus of the MS2 coat protein [27,40]. FP8 is a short linear epitope, solvent accessible and recognized by bnAbs at the N-terminus of HIV-1 gp41 [12,16]. Correspondingly, the FP8 sequence was inserted at the N-terminus of the MS2 single-chain dimer, reasoning that display of FP8 in this context would be most similar to its native conformation. In order to produce recombinant MS2-FP8 VLPs, the FP8 sequence was genetically inserted at the N-terminus of the single-chain dimer of MS2 coat protein (shown schematically in Figure 1b). The recombinant VLP comprises 90 single-chain dimers of MS2 coat protein, which self-assemble into FP8-displaying MS2 VLP in vivo in *E. coli*. Thus, recombinant MS2-FP8 VLPs are predicted to display exactly 90 copies of the FP8 peptide per particle.

**Figure 2:**
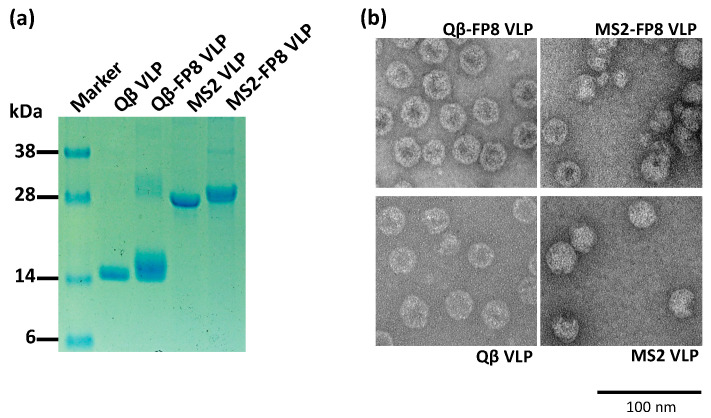
Characterization of conjugated (Qβ-FP8) and recombinant (MS2-FP8) VLPs. (**a**) SDS-PAGE analysis of proteins following the conjugation of FP8 peptides to microbially synthesized Qβ bacteriophage VLPs and the downstream processing of an *E. coli* expressed and in vivo assembled MS2-FP8 VLPs. The dominant bands on the gel correspond to product of the expected size. The ladder of bands in the Qß-FP8 VLP lane reflect individual copies of coat protein modified with 0, 1, 2, or more copies of the FP8 peptide. An unmodified version of this gel is shown in Figure S1. (**b**) Transmission electron micrograms (TEM) of conjugated (Qβ-FP8) and recombinant (MS2-FP8) VLPs in comparison to unconjugated and unmodified microbially synthesized Qβ and MS2 VLPs, respectively. Scale bar is 100 nm.

Microbially synthesized Qβ bacteriophage VLPs have been previously modified to display diverse antigens using a chemical crosslinker, in which peptides can be attached to exposed lysine residues that are abundantly displayed on the VLP surface [22,41]. Figure 1a illustrates this chemical technique for the construction of an FP8-displaying Qβ-VLP. The 8 amino acid FP8 epitope was chemically synthesized to include a C-terminal conjugation tag consisting of three glycines and a cysteine (FP8-*GGGC*). The synthetic FP8 peptides containing free cysteines were coupled to Qβ-VLPs using a bifunctional cross-linker (SMPH), yielding conjugated particles (Qß-FP8 VLPs). An analysis of conjugation efficiency by SDS-PAGE indicated that this technique resulted in an average of >200 FP8 peptides displayed per Qβ-VLP (Figure 2a, Lane 3).

Figure 2a shows SDS-PAGE analysis of purified Qβ-FP8 and MS2-FP8 VLPs. The lanes in Figure 2a demonstrate successful conjugation of FP8 to the Qβ coat protein (Lane 3) and insertion of FP8 at the N-terminus of the single-chain dimer of MS2 coat protein (Lane 5). As shown by SDS-PAGE analysis (Figure 2a), conjugated Qβ-FP8 VLPs and recombinant purified MS2-FP8 VLPs were over 90% pure. Centrifugation of conjugation reaction products using ultrafilters removed excess SMPH crosslinkers and FP8 peptides, yielding pure Qβ-FP8 VLPs. Selective salting-out precipitation of target proteins from *E. coli* cell lysates, with a polishing SEC step, resulted in the separation of recombinant in vivo assembled MS2-FP8 VLPs from most host cell protein contaminants.

**Figure 3:**
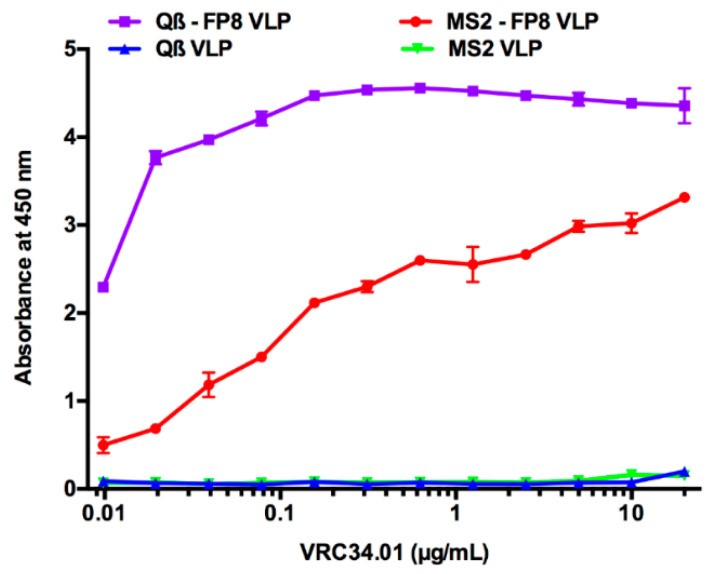
The FP8 peptide epitope is displayed on Qβ-VLPs and MS2-VLPs, as shown by ELISA. Qβ-FP8 VLP, MS2-FP8 VLP, or wild type (Qβ and MS2) VLPs (negative controls) were plated and probed with 2-fold dilutions of VRC34.01 bnAb. Reactivity of VRC34.01 confirms display of FP8 peptides on the surface of both VLPs. Absorbance values at each dilution point show the mean ± S.D of replicate wells (n = 2).

To further characterize the FP8-displaying VLPs, VLPs were visualized by transmission electron microscopy (TEM) and also assessed VRC34.01 binding by ELISA. TEM micrographs of the conjugated (Qβ-FP8), the recombinant (MS2-FP8) and the unmodified (Qβ and MS2) VLPs are shown in Figure 2b. Under TEM, Qβ-FP8, and MS2-FP8 VLPs were similar in morphology to the corresponding unmodified (Qβ and MS2) VLPs, confirming the particulate and multivalent nature of the FP8-displaying VLPs. Strong binding reactivity of Qβ-FP8 VLP and MS2-FP8 VLP with VRC34.01 (Figure 3) using direct ELISA confirmed display of the FP8 epitope on the surface of VLPs. The stronger binding reactivity of Qβ-FP8 VLP is likely due to higher valency of FP8 peptides on the Qß-FP8 VLPs (>200 copies) versus the MS2-FP8 VLPs (90 copies). Thus, the peptide insertion and conjugation strategies were successful approaches for constructing FP8-displaying MS2 and Qβ VLPs.

### 3.2. Immunogenicity of FP8-VLPs

Much effort has been directed to design immunogens and to find the optimal immunization strategies in order to efficiently elicit HIV neutralizing antibodies against discrete epitopes. The RV144 vaccine clinical trial, which showed modest protection from HIV infection, employed a prime/boost-based vaccination design with multiple immunizations [42]. Other prime-boost vaccination approaches have also shown potential in preclinical and clinical development of HIV vaccines [43]. However, the ability to successfully elicit neutralizing antibodies is likely dependent on multiple factors, including the choice and order of priming-and boosting-immunogens. Here, the immunogenicity of unadjuvanted FP8-VLP vaccines were compared in mice using both homologous and heterologous prime-boost regimens. Some groups of mice were also boosted with full-length trimeric Env protein (BG505 DS-SOSIP), because previous studies have shown that boosts with this antigen can amplify FP8-targeted antibody responses [15]. As controls, mice were immunized with wild-type VLPs.

**Figure 4:**
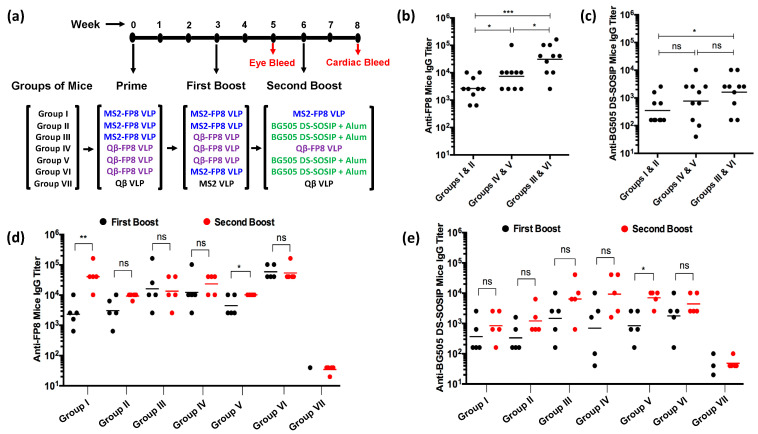
Immune responses in mice vaccinated with FP8-VLPs. (**a**) Immunization regimen. Groups of 5 mice were given three immunizations with FP8-VLPs (Groups I-VI) or control VLPs (Group VII). Mice were immunized at weeks 0 (prime), 3 (first boost), and 6 (second boost). At the second boost, some groups were immunized with native HIV-1 trimer (BG505 DS-SOSIP) plus Alum adjuvant. Serum collections occurred two weeks after the first boost [week 5] and the second boost [week 8]. Evaluation of the effect of homologous vs heterologous prime-boost regimen on the level of (**b**) FP8- and (**c**) full-length HIV-1 Envelope trimer (BG505 DS-SOSIP)-specific total IgG titers, which were measured by ELISA using week 5 immune sera. Data were combined for each MS2-FP8VLP, Qβ-FP8 VLP homologous-, and MS2-FP8 VLP/Qβ-FP8 VLP heterologous-prime boosting immunizations. Each dot represents the IgG titer from a single mouse. Lines represent geometric means (n = 10 per group). *p* values were calculated by unpaired two-tailed t test. *, *p* < 0.05, **, *p* < 0.01, ***, *p* < 0.001. (**d**) Anti-FP8 and (**e**) Anti-BG505 DS-SOSIP IgG end-point dilution antibody titers after the first boost (black dots) and second boost (red dots) were measured by ELISA analysis of sera. Lines represent geometric means (n = 5 per group). Statistical analysis of antibody titers after the first and second boost is presented. * *p* < 0.05 and ** *p* < 0.01 indicate that there is a significant difference between the antibody titers of each group after the first and second boost, *ns* indicates no significant difference.

Figure 4a outlines the vaccination schedule and the immunogens used at prime, first boost, and second boost in each of seven groups of mice. Mice sera were obtained after the first and second boosts and antibodies against the FP8 peptide and full-length BG505 DS-SOSIP were measured by ELISA. First, to examine antibody responses elicited by FP8-VLP immunization alone, the FP8- and BG505 DS-SOSIP-specific IgG titers were measured following two immunizations with MS2-FP8 VLP (Groups I and II), Qβ-FP8 VLP (Groups IV and V) and the heterologous prime-boost groups (Groups III and VI). All of the vaccinated groups produced high titer IgG, which recognized the FP8 peptide (Figure 4b). The heterologous prime-boost regimen of FP8-VLPs (Group III and VI) induced a mean anti-FP8 antibody endpoint titer of more than 10^4^ after the first boost, significantly higher than the homologous prime-boost immunizations with MS2-FP8 VLPs (more than 13-fold higher; *p* < 0.001) or Qβ-FP8 VLPs (more than 3-fold higher; *p* < 0.05) (Figure 4b). In addition, two doses of Qβ-FP8 VLPs induced anti-FP8 antibody levels that were significantly higher than the groups immunized with MS2-FP8 VLPs (*p* < 0.05), indicating that increase antibody titers are likely due to the higher density of FP8 peptides (>200 versus 90) on the surface of Qβ VLPs. Anti-FP8 antibodies elicited by each of the experimental groups were also capable of recognizing full-length BG505 DS-SOSIP trimers (shown in Figure 4c). Similar to the FP8-specific responses, the groups that received a heterologous prime-boost regimen had the highest BG505 DS-SOSIP-specific IgG titers. These results were not unexpected, since several other studies have shown that heterologous immunizations can be more effective than homologous immunizations [43,44]. The specific mechanisms for the improved efficacy of the heterologous prime-boost vaccination are unknown, but it is possible that using multiple VLP platforms reduces the possibility that antibodies against the platform could interfere with responses against the FP8 peptide, mitigating the phenomenon referred to as carrier-mediated suppression [45]. However, there is no evidence that carrier-mediated suppression reduces antibody responses to antigens displayed on VLPs; for example, suppression has not been observed in human clinical trials of Qβ bacteriophage VLP-based vaccines [32,33,34,46]. Nevertheless, the present study demonstrates the effectiveness of a prime-boost approach using heterologous non-cross-reactive bacteriophage VLP carriers (Qβ and MS2) for enhancing FP8-specific antibody responses.

Although VRC34.01 recognizes a linear epitope, glycosylation events in envelope outside of the FP8 sequence can modulate its binding to envelope [12,16]. Other FP8-based vaccine studies have demonstrated that boosting with native envelope can fine-tune the antibody responses and result in enhanced neutralization breadth of FP8-elicited antibodies [3,14,15,16,17]. To assess the impact of boosting with trimer, some FP8-VLP immunized groups (shown in Figure 4a) received an additional boost with adjuvanted BG505 DS-SOSIP. Antibody responses against FP8 (Figure 4d, red dots) and BG505 DS-SOSIP (Figure 4e, red dots) were measured by ELISA to compare antibody titers prior to and following the second boost. All of the groups immunized with FP8 vaccines produced high titer anti-FP8 (*p* < 0.0001) and anti-SOSIP (*p* < 0.01) IgG in comparison to the negative control (Group VII). Boosting with BG505 DS-SOSIP did not significantly increase the level of anti-FP8 antibody titer in comparison to its level after the first boost (Figure 4d, red dots vs black dots, groups II, III, & VI). Boosting with BG505 DS-SOSIP, however, did result in an increase in anti-BG505 DS-SOSIP antibody titers. Anti-FP8 antibodies elicited by the groups of mice that only received three homologous boosts of MS2-FP8 or Qβ-FP8 VLPs (groups I and IV) were also capable of binding to BG505 DS-SOSIP.

### 3.3. FP8-VLPs Elicit HIV-1 Neutralizing Antibodies

**Figure 5:**
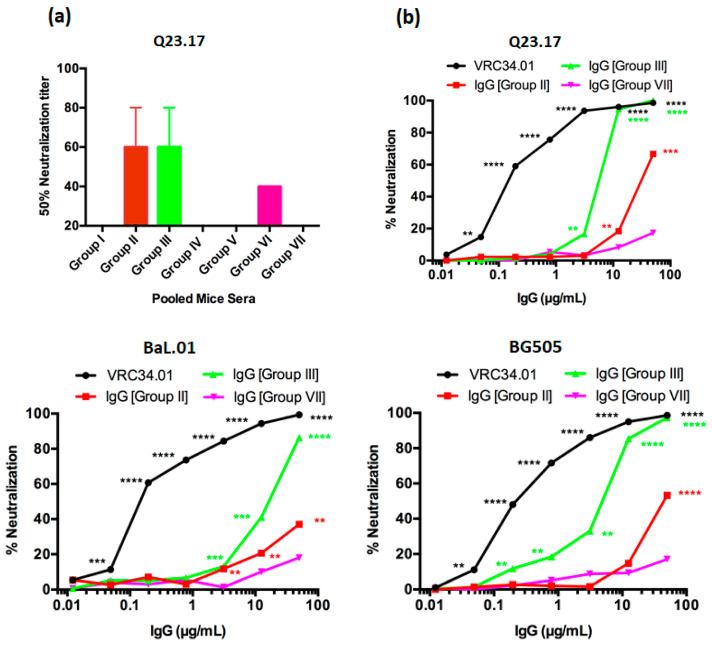
Neutralization activity of pooled sera (**a**) or pooled purified IgG (**b**) from FP8-VLP immunized mice. In vitro neutralization activity was determined using TZM-bl cells infected with Q23.17 (Clade A), BG505 (Clade A) and BaL.01 (Clade B) HIV-1 virus. (a) Neutralizing activity of pooled week 8 sera obtained from BALB/c mice immunized with various prime-boost-boost compositions containing FP8-VLPs alone or FP8-VLPs and BG505 DS-SOSIP was determined against Q23.17. The neutralization titer is expressed as the reciprocal of the highest serum dilution reducing cell infection by 50%. The lowest dilution tested was 1:20. (b) The viral neutralization activity of dilutions of purified serum IgG obtained from FP8-VLPs immunized mice with different prime boosting regimens was determined against Q23.17, BG505, and BaL.01, and is presented as percentage neutralization. VRC34.01 (positive) and IgG from Qβ/MS2 VLP immunized mice (negative) were used as controls. *p* values were calculated by paired two-tailed t test. **, *p* < 0.01, ***, *p* < 0.001, ****, *p* < 0.0001.

Production of high levels of FP8-specific antibodies in mice immunized with FP8-VLPs, and their binding reactivity to BG505 DS-SOSIP are potentially indicative of FP8-specific HIV-1 neutralizing activity. The ability of the anti-FP8 peptide sera to prevent viral infection in vitro was initially examined by a TZM-bl virus neutralization assay, using pooled sera from each group of mice against the Clade A primary HIV-1 isolate Q23.17. Q23.17, a Tier 2 isolate, was chosen because this virus is sensitive to neutralization by the positive control mAb, VRC34.01 [3]. As shown in Figure 5, sera from several of the groups of FP8-VLP immunized mice could neutralize HIV-1 Q23.17. The anti-FP8 sera from Group II and Group III mice were able to inhibit infection by 50% at a 1:80 dilution, and Group VI mice sera showed 50% neutralization at a 1:40 dilution (Figure 5a). Immune sera obtained from Group I, Group IV, and Group V mice had weak neutralizing activity, but neutralization did not reach the 50% threshold at the lowest dilution tested.

Next, the potency of purified IgG from the two groups (Group II and Group III) of mice with the strongest neutralizing activity against Q23.17 was tested against three prototype HIV-1 isolates. As shown in Figure 5b, purified serum IgGs could neutralize prototype clade A (Q23.17 & BG505; both Tier 2) and clade B (BaL.01; Tier 1b) viruses. In particular, IgG isolated from Group III mice, which received a heterologous prime/boost/boost regimen, had potent neutralizing activity, exhibiting nearly complete neutralization of isolates Q23.17 and BG505 at a concentration of 12.5 µg/mL (Figure 5b). Purified IgG from Group II also neutralized HIV-1, albeit with lower potency. Taken together, these data highlight the importance of heterologous boosting in generating FP8-specific antibodies with HIV-1 neutralizing activity.

Interestingly, sera from some immunized groups with high anti-FP8 antibody levels (such as in Group I) or high titer antibodies against BG505 DS-SOSIP of (such as in Group IV) did not neutralize HIV-1. The lack of correlation between the high level of anti-FP8 titers and the elicited neutralization activity of the mice sera suggests a critical parameter may be recognition of a specific conformation of the peptide, or, perhaps, appropriate affinity maturation of the responses, but most likely not the overall titer of reactive antibodies. Affinity maturation often increases the affinity, avidity, and neutralization activity of antibodies through multiple rounds of somatic hypermutation and selection in the germinal center [47,48,49]. Recent studies have used innovative vaccine design approaches and immunization strategies to trigger B cell precursors expressing germline receptors and then direct affinity maturation toward HIV-1 broad neutralizing antibodies [1,49,50,51]. Xu et al., [3] demonstrated that an immunogen design based on FP8 linked to KLH and an immunization strategy, involving FP8-KLH priming and Env-trimer boosting, elicited FP8-directed cross-clade neutralizing antibodies in mice, guinea pigs, and non-human primates. Multiple FP8-KLH primes and Env-trimer boosts increased FP8-directed cross-clade HIV neutralization breadth [14,52]. These published results [3,15,52] and the results described in this study highlight the importance of the prime/boost compositions and immunization strategies and indicate that a sequence of immunizations with different immunogens may be a key to guide affinity maturation of FP8-directed bnAbs. In addition, well validated genetic and structural approaches for identification and characterization of neutralizing antibody lineages in vaccinated animals [3,14], open the opportunity to find an optimal immunogen design and immunization strategy that can induce the broadest FP8-directed neutralization.

## 4. Conclusions

FP8, a site of vulnerability on the HIV-1 Env glycoprotein and a target for infection-elicited bnAbs, has become a promising target for HIV-1 vaccine design. Our aim was to develop new HIV vaccine candidates by displaying the FP8 peptide on the surface of microbially synthesized RNA bacteriophage VLPs and to find an optimal prime-boost immunization strategy for eliciting high titer FP8-directed HIV-1 neutralizing antibodies. Bacteriophage VLPs, which display the FP8 peptide in a multivalent fashion, were produced using two techniques, chemical conjugation, and genetic insertion. Both approaches yielded FP8-displaying VLPs, which reacted strongly with an FP8-binding bnAb, VRC34.01, and, in mice, elicited antibodies that bound to native Env trimer. The FP8-VLPs were tested in different prime-boost regimens to elicit FP8-specific HIV-1 neutralization activity in mice. Immunization with MS2-FP8 VLP prime, Qβ-FP8 VLP first boost and alum adjuvanted BG505 DS-SOSIP second boost induced the most potent HIV-1 neutralizing antibody responses, highlighting the importance of a heterologous boosting regimen. The results in this study demonstrate that an approach combining the peptide display platform based on the RNA bacteriophage VLPs and a prime-boost-boost immunization strategy allowed the elicitation of HIV-1 neutralizing antibodies with activity against a small panel of HIV-1 isolates in mice. While there are limitations to our study—we did not assess the breadth of HIV-1 neutralizing activity and the mechanistic contributions of SOSIP boosting has yet to be determined—these promising results warrant further studies to explore the full potential of FP8-VLP vaccine designs with multiple prime-boost compositions for the elicitation of the broadest FP8-directed neutralizing antibody responses. Further study is also needed to identify and characterize FP8-directed broad neutralizing antibody lineages in vaccinated animals, as well as to explore different adjuvants and multiple Env trimer boosts to increase neutralization titer and breadth.

## Supplementary Materials

The following are available online at https://www.mdpi.com/2076-393X/8/4/765/s1, Figure S1: Uncropped gel shown in Figure 2a.

## References

[1] Escolano A., Steichen J.M., Dosenovic P., Kulp D.W., Golijanin J., Sok D., Freund N.T., Gitlin A.D., Oliveira T., Araki T. (2016). Sequential Immunization Elicits Broadly Neutralizing Anti-HIV-1 Antibodies in Ig Knockin Mice. Cell.

[2] Saunders K.O., Verkoczy L.K., Jiang C., Zhang J., Parks R., Chen H., Housman M., Bouton-Verville H., Shen X., Trama A.M. (2017). Vaccine Induction of Heterologous Tier 2 HIV-1 Neutralizing Antibodies in Animal Models. Cell Rep..

[3] Xu K., Acharya P., Kong R., Cheng C., Chuang G.Y., Liu K., Louder M.K., O’Dell S., Rawi R., Sastry M. (2018). Epitope-based vaccine design yields fusion peptide-directed antibodies that neutralize diverse strains of HIV-1. Nat. Med..

[4] Dashti A., DeVico A.L., Lewis G.K., Sajadi M.M. (2019). Broadly Neutralizing Antibodies against HIV: Back to Blood. Trends Mol. Med..

[5] Liao H.X., Lynch R., Zhou T., Gao F., Alam S.M., Boyd S.D., Fire A.Z., Roskin K.M., Schramm C.A., Zhang Z. (2013). Co-evolution of a broadly neutralizing HIV-1 antibody and founder virus. Nature.

[6] Bonsignori M., Zhou T., Sheng Z., Chen L., Gao F., Joyce M.G., Ozorowski G., Chuang G.Y., Schramm C.A., Wiehe K. (2016). Maturation Pathway from Germline to Broad HIV-1 Neutralizer of a CD4-Mimic Antibody. Cell.

[7] Doria-Rose N.A., Schramm C.A., Gorman J., Moore P.L., Bhiman J.N., DeKosky B.J., Ernandes M.J., Georgiev I.S., Kim H.J., Pancera M. (2014). Developmental pathway for potent V1V2-directed HIV-neutralizing antibodies. Nature.

[8] Landais E., Murrell B., Briney B., Murrell S., Rantalainen K., Berndsen Z.T., Ramos A., Wickramasinghe L., Smith M.L., Eren K. (2017). HIV Envelope Glycoform Heterogeneity and Localized Diversity Govern the Initiation and Maturation of a V2 Apex Broadly Neutralizing Antibody Lineage. Immunity.

[9] Bonsignori M., Kreider E.F., Fera D., Meyerhoff R.R., Bradley T., Wiehe K., Alam S.M., Aussedat B., Walkowicz W.E., Hwang K.K. (2017). Staged induction of HIV-1 glycan-dependent broadly neutralizing antibodies. Sci. Transl. Med..

[10] MacLeod D.T., Choi N.M., Briney B., Garces F., Ver L.S., Landais E., Murrell B., Wrin T., Kilembe W., Liang C.H. (2016). Early Antibody Lineage Diversification and Independent Limb Maturation Lead to Broad HIV-1 Neutralization Targeting the Env High-Mannose Patch. Immunity.

[11] Huang J., Ofek G., Laub L., Louder M.K., Doria-Rose N.A., Longo N.S., Imamichi H., Bailer R.T., Chakrabarti B., Sharma S.K. (2012). Broad and potent neutralization of HIV-1 by a gp41-specific human antibody. Nature.

[12] Kong R., Xu K., Zhou T., Acharya P., Lemmin T., Liu K., Ozorowski G., Soto C., Taft J.D., Bailer R.T. (2016). Fusion peptide of HIV-1 as a site of vulnerability to neutralizing antibody. Science.

[13] Kelsoe G., Haynes B.F. (2018). What Are the Primary Limitations in B-Cell Affinity Maturation, and How Much Affinity Maturation Can We Drive with Vaccination? Breaking through Immunity’s Glass Ceiling. Cold Spring Harb. Perspect. Biol..

[14] Dingens A.S., Acharya P., Haddox H.K., Rawi R., Xu K., Chuang G.Y., Wei H., Zhang B., Mascola J.R., Carragher B. (2018). Complete functional mapping of infection- and vaccine-elicited antibodies against the fusion peptide of HIV. PLoS Pathog..

[15] Kong R., Duan H., Sheng Z., Xu K., Acharya P., Chen X., Cheng C., Dingens A.S., Gorman J., Sastry M. (2019). Antibody Lineages with Vaccine-Induced Antigen-Binding Hotspots Develop Broad HIV Neutralization. Cell.

[16] Yuan M., Cottrell C.A., Ozorowski G., van Gils M.J., Kumar S., Wu N.C., Sarkar A., Torres J.L., de Val N., Copps J. (2019). Conformational Plasticity in the HIV-1 Fusion Peptide Facilitates Recognition by Broadly Neutralizing Antibodies. Cell Host Microbe.

[17] Kumar S., Sarkar A., Pugach P., Sanders R.W., Moore J.P., Ward A.B., Wilson I.A. (2019). Capturing the inherent structural dynamics of the HIV-1 envelope glycoprotein fusion peptide. Nat. Commun..

[18] Keating G.M., Noble S. (2003). Recombinant hepatitis B vaccine (Engerix-B): A review of its immunogenicity and protective efficacy against hepatitis B. Drugs.

[19] Schiller J., Lowy D. (2018). Explanations for the high potency of HPV prophylactic vaccines. Vaccine.

[20] Wu T., Li S.W., Zhang J., Ng M.H., Xia N.S., Zhao Q. (2012). Hepatitis E vaccine development: A 14 year odyssey. Hum. Vaccines Immunother..

[21] Chackerian B. (2007). Virus-like particles: Flexible platforms for vaccine development. Expert Rev. Vaccines.

[22] Frietze K.M., Peabody D.S., Chackerian B. (2016). Engineering virus-like particles as vaccine platforms. Curr. Opin. Virol..

[23] Lua L.H., Connors N.K., Sainsbury F., Chuan Y.P., Wibowo N., Middelberg A.P. (2014). Bioengineering virus-like particles as vaccines. Biotechnol. Bioeng..

[24] Mohsen M.O., Zha L., Cabral-Miranda G., Bachmann M.F. (2017). Major findings and recent advances in virus-like particle (VLP)-based vaccines. Semin. Immunol..

[25] Pumpens P., Renhofa R., Dishlers A., Kozlovska T., Ose V., Pushko P., Tars K., Grens E., Bachmann M.F. (2016). The True Story and Advantages of RNA Phage Capsids as Nanotools. Intervirology.

[26] Peabody D.S., Manifold-Wheeler B., Medford A., Jordan S.K., do Carmo Caldeira J., Chackerian B. (2008). Immunogenic display of diverse peptides on virus-like particles of RNA phage MS2. J. Mol. Biol..

[27] Tumban E., Peabody J., Tyler M., Peabody D.S., Chackerian B. (2012). VLPs displaying a single L2 epitope induce broadly cross-neutralizing antibodies against human papillomavirus. PLoS ONE.

[28] O’Rourke J.P., Peabody D.S., Chackerian B. (2015). Affinity selection of epitope-based vaccines using a bacteriophage virus-like particle platform. Curr. Opin. Virol..

[29] Jegerlehner A., Storni T., Lipowsky G., Schmid M., Pumpens P., Bachmann M.F. (2002). Regulation of IgG antibody responses by epitope density and CD21-mediated costimulation. Eur. J. Immunol..

[30] Crossey E., Amar M.J.A., Sampson M., Peabody J., Schiller J.T., Chackerian B., Remaley A.T. (2015). A cholesterol-lowering VLP vaccine that targets PCSK9. Vaccine.

[31] Maphis N.M., Peabody J., Crossey E., Jiang S., Jamaleddin Ahmad F.A., Alvarez M., Mansoor S.K., Yaney A., Yang Y., Sillerud L.O. (2019). Qss Virus-like particle-based vaccine induces robust immunity and protects against tauopathy. NPJ Vaccines.

[32] Kundig T.M., Senti G., Schnetzler G., Wolf C., Prinz Vavricka B.M., Fulurija A., Hennecke F., Sladko K., Jennings G.T., Bachmann M.F. (2006). Der p 1 peptide on virus-like particles is safe and highly immunogenic in healthy adults. J. Allergy Clin. Immunol..

[33] Maurer P., Bachmann M.F. (2010). Immunization against angiotensins for the treatment of hypertension. Clin. Immunol..

[34] Maurer P., Jennings G.T., Willers J., Rohner F., Lindman Y., Roubicek K., Renner W.A., Muller P., Bachmann M.F. (2005). A therapeutic vaccine for nicotine dependence: Preclinical efficacy, and Phase I safety and immunogenicity. Eur. J. Immunol..

[35] Chackerian B., Caldeira Jdo C., Peabody J., Peabody D.S. (2011). Peptide epitope identification by affinity selection on bacteriophage MS2 virus-like particles. J. Mol. Biol..

[36] Chackerian B., Rangel M., Hunter Z., Peabody D.S. (2006). Virus and virus-like particle-based immunogens for Alzheimer’s disease induce antibody responses against amyloid-beta without concomitant T cell responses. Vaccine.

[37] Tumban E., Peabody J., Peabody D.S., Chackerian B. (2011). A pan-HPV vaccine based on bacteriophage PP7 VLPs displaying broadly cross-neutralizing epitopes from the HPV minor capsid protein, L2. PLoS ONE.

[38] Kwon Y.D., Pancera M., Acharya P., Georgiev I.S., Crooks E.T., Gorman J., Joyce M.G., Guttman M., Ma X., Narpala S. (2015). Crystal structure, conformational fixation and entry-related interactions of mature ligand-free HIV-1 Env. Nat. Struct. Mol. Biol..

[39] Georgiev I.S., Joyce M.G., Yang Y., Sastry M., Zhang B., Baxa U., Chen R.E., Druz A., Lees C.R., Narpala S. (2015). Single-Chain Soluble BG505.SOSIP gp140 Trimers as Structural and Antigenic Mimics of Mature Closed HIV-1 Env. J. Virol..

[40] Zhai L., Peabody J., Pang Y.S., Schiller J., Chackerian B., Tumban E. (2017). A novel candidate HPV vaccine: MS2 phage VLP displaying a tandem HPV L2 peptide offers similar protection in mice to Gardasil-9. Antivir. Res..

[41] Jennings G.T., Bachmann M.F. (2008). The coming of age of virus-like particle vaccines. Biol. Chem..

[42] Rerks-Ngarm S., Pitisuttithum P., Nitayaphan S., Kaewkungwal J., Chiu J., Paris R., Premsri N., Namwat C., de Souza M., Adams E. (2009). Vaccination with ALVAC and AIDSVAX to prevent HIV-1 infection in Thailand. N. Engl. J. Med..

[43] Lu S. (2009). Heterologous prime-boost vaccination. Curr. Opin. Immunol..

[44] Ratto-Kim S., Currier J.R., Cox J.H., Excler J.L., Valencia-Micolta A., Thelian D., Lo V., Sayeed E., Polonis V.R., Earl P.L. (2012). Heterologous prime-boost regimens using rAd35 and rMVA vectors elicit stronger cellular immune responses to HIV proteins than homologous regimens. PLoS ONE.

[45] Kardani K., Bolhassani A., Shahbazi S. (2016). Prime-boost vaccine strategy against viral infections: Mechanisms and benefits. Vaccine.

[46] Cornuz J., Zwahlen S., Jungi W.F., Osterwalder J., Klingler K., van Melle G., Bangala Y., Guessous I., Muller P., Willers J. (2008). A vaccine against nicotine for smoking cessation: A randomized controlled trial. PLoS ONE.

[47] Bannard O., Cyster J.G. (2017). Germinal centers: Programmed for affinity maturation and antibody diversification. Curr. Opin. Immunol..

[48] Doria-Rose N.A., Joyce M.G. (2015). Strategies to guide the antibody affinity maturation process. Curr. Opin. Virol..

[49] Havenar-Daughton C., Abbott R.K., Schief W.R., Crotty S. (2018). When designing vaccines, consider the starting material: The human B cell repertoire. Curr. Opin. Immunol..

[50] Andrabi R., Bhiman J.N., Burton D.R. (2018). Strategies for a multi-stage neutralizing antibody-based HIV vaccine. Curr. Opin. Immunol..

[51] Briney B., Sok D., Jardine J.G., Kulp D.W., Skog P., Menis S., Jacak R., Kalyuzhniy O., de Val N., Sesterhenn F. (2016). Tailored Immunogens Direct Affinity Maturation toward HIV Neutralizing Antibodies. Cell.

[52] Cheng C., Xu K., Kong R., Chuang G.Y., Corrigan A.R., Geng H., Hill K.R., Jafari A.J., O’Dell S., Ou L. (2019). Consistent elicitation of cross-clade HIV-neutralizing responses achieved in guinea pigs after fusion peptide priming by repetitive envelope trimer boosting. PLoS ONE.

