# Supplementary material for: Virus-Like Particle Based Vaccines Elicit Neutralizing Antibodies against the HIV-1 Fusion Peptide
Source: Vaccines (Basel). 2020 Dec 15;8(4):765. doi: 10.3390/vaccines8040765 (PMC7765226; doi:10.3390/vaccines8040765)
Supplement: Supplementary file 1 [file vaccines-08-00765-s001.pdf]

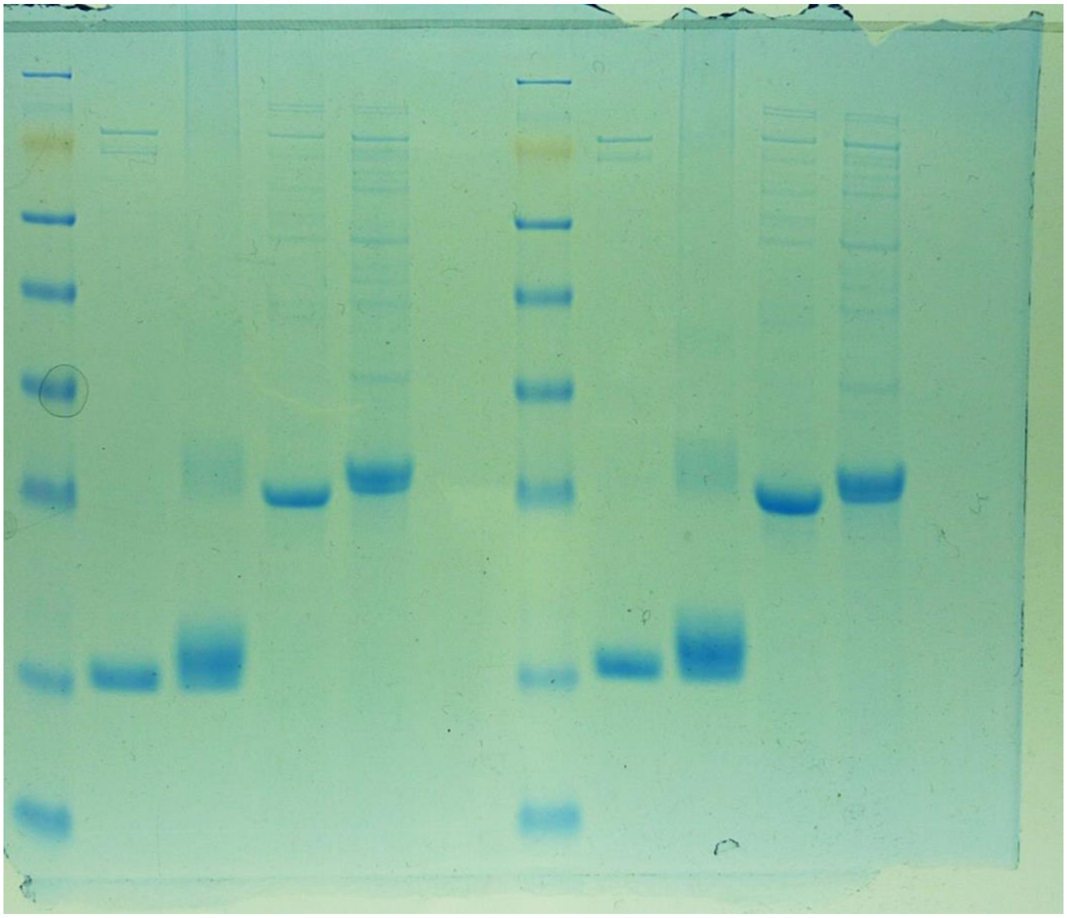

Figure S1. Unmodified SDS-PAGE gel used as a source of the data shown in Figure 2a.

Lanes 1 and 7 contain molecular weight markers (Invitrogens SeeBlue Plus2 Prestained Markers). Lanes 2 and 8 contain unmodified Qß VLPs, Lanes 3 and 9 contain Qß-FP8 VLPs, Lanes 4 and 10 contain unmodified MS2 VLPs, and Lanes 4 and 11 contains MS2-FP8 VLPs. Lanes 7-11 were included in Figure 2a.
